# Assistance and Assistants

**Published:** 1895-12

**Authors:** C. A. Brackett

**Affiliations:** Newport, B. I.


### ASSISTANCE AND ASSISTANTS.[1]

BY C. A. BRACKETT, D.M.D., NEWPORT, R. I.

In the old days of negro slavery in a portion of our land it was said that differing characteristics between people of different sections might be described thus: A Southerner never does for himself anything which another can do for him, and he never does alone that about which he may have help. The New-Englander never asks another to do for him that which he can do for himself, and he does not have help in doing that which he can do alone. In the old order of things, with people of whom we speak as belonging to the old school, these descriptions were not far from having a good foundation in truth; but time and circumstances have brought changes in the way of doing things to all.

I am myself of New England parentage, the descendant of a stock that had all along been under the necessity of hard work,—harder than we of to-day always realize and remember,—and they brought up their children to work, to work all day, and to work every day, and to work quite after the defined New England fashion. I shall never forget the jealousy which I had when ten years of age of an older boy in the neighborhood who had been hired to help in cutting up the wood-pile which I wished to cut alone.

I got also by heredity, example, and precept other tendencies and other training which included in detail much guidance in regard to the practical affairs of life. I was taught that I should mind my own business; that I should avoid partnership relations; that I should seek to keep my own affairs in my own control, and that I should be cautious about becoming responsible for the doings of others. However poorly I may have learned the excellent lessons put before me, I shall never cease to be grateful to the best

---

[1] Read before the American Academy of Dental Science, Boston, April 3, 1895.

of fathers and the best of mothers who gave me the substance of all that there may be in me of good. And what I have said of my own case finds doubtless its counterpart in the experience of nearly every one seated about this table to-night. It is the story of a large part of those who have made their lives a credit to themselves, an honor to their parentage, and a service to the world. The spirit of such training can never become superannuated or shown to be fallacious. Superficially, it may seem not quite in harmony with modern methods; essentially, all that is good in each is in entire accord with the other.

This congruity I did not at once see. When I came under the necessity of being the manager of my own affairs, I had a little time of seeking to do everything that I could with my own hands. That time I do not regret. On the contrary, I rejoice to have had the experience just as it was. It was most appropriate to the circumstances of the time; it stimulated my efforts, extended my resources, cultivated my self-reliance; was often that necessity which becomes the mother of invention, and it made the proper foundation upon which to gradually develop the plan of putting out of my own hands that which might be done equally well by others. Twenty-two years I have been a practitioner of dentistry. In the last twenty years of that time I have had help in my work.

On the title-page or in the preface of almanacs we are accustomed to find a statement to the effect that the calculations which follow are accurate for some particular place, as Boston, and that they may be made to serve for a considerable surrounding territory, as New England. My experience has, of course, been very largely dependent upon circumstances of time and place, and greatly modified by those circumstances. For other circumstances the calculation would need to be varied, or perhaps could not be made to serve at all.

When I located in Newport, there was room for me and work for me to do. The work was begun with certain ideas concerning it. Some of those ideas have been proved erroneous; others have become in the passing years more and more positive convictions. Newport is a small city; and it has seemed to me that in at least two prominent particulars dental practice in a small place should differ from that which is permissible, not to say advisable, in a large city. One of these particulars has reference to the subdivision of practice, the making and following of a specialty within the specialty. In the large city the dentist may do no extracting, or he may do nothing except extracting. He may give his whole attention to the general care of children's teeth, to the correction of irregularities, the conservation of adult's natural teeth, the insertion of artificial teeth, crown- and bridge-work, or the practice of oral surgery, with the probable consequence that the entire community has its dental needs the better served for his so doing. Again, in the large city, where there are many practitioners, the man of experience, of superior abilities, extending reputation, and rapidly growing practice may increase his fees beyond the ordinary, or beyond what they were in his beginning, and this, if properly and honestly done, without injustice to any one. There are inexorable limitations to the amount of work which one man may do in his life, and it is legitimate that the compensation for that work should be influenced somewhat by the same laws of supply and demand which dominate values in the commercial world. The express train would cease to be an express train, or to possess the advantages of an express train, if it went at the speed of an accommodation train and stopped at every station. The railroad system which stopped every train at every station would serve but poorly the convenience of its patrons. So in the large city, the dentist upon whom extraordinary demands are made, and who can command extraordinary compensation for his genuinely skilled and superior services, may be not only justified in avowedly making his fees high, but he may find it his duty to do so in order to so limit his work that his physical and mental condition shall be always such that his patients may invariably receive the superior service for which they pay. Further, he may by such course, without making hardship for any not well able to pay for his service, be aiding in justice to his dental neighbor, equally faithful, but younger, less experienced, or less well known.

I am unable to mention this subject of fees without an expression of caution to men, and especially to young men, that they do not put effect in the place of cause, and be hasty and arbitrary in demanding the high fee, thinking more of that than they do of first attaining the superiority of service. There are needed many more accommodation trains than express trains, and we should all seek to avoid mistaking the calling with which we are called. In the small town the plan of indiscriminately raising the fees in order to lessen the volume of practice is not usually a thing to be accomplished without imposing hard conditions upon some dwellers in the community.

While, perhaps, having a smaller proportion of extremely poor people than most places, Newport does present an exceptionally great range in the material means and the advantages which means command, of its population. A proper share of all these classes of people rightfully expect that their needs for dental service shall be met by local practitioners, and they are so met. A moment's thought will show that a practice of such range of demands is more complicated to manage with satisfaction to all concerned than is one more nearly uniform in means, advantages, standing, desires, and ways of doing things. In the complex practice assistance seems to have particular adaptation, and actually independent operative assistance may probably be more readily made helpful than in an exclusively high-class practice.

Except it may be in regions out of the range of the intimate acquaintance of those who are assembled here, the taking of students—that is, entire novices—into offices, with the double object of the principal gaining assistance in his work and the young man being made a dentist, is happily almost entirely a thing of the past. Some of us can almost remember the time when the student of general medicine got his instruction, both theoretical and practical, largely from a private preceptor. Every one of us is cognizant of the fact that even up to the present a similar plan has been followed in the study of the law, but in the law the plan is fast passing out. It is to-day almost forgotten that it ever had place in the study of medicine. Such of it as continues in dentistry can advantageously be only to supplement and make more perfect the teaching of the schools. While it is to be maintained that the schools are right in requiring the attendance of their pupils through three full school years, it is to be admitted that there are certain graces that have their acquirement and cultivation greatly favored by a little experience in the atmosphere of a refined private office.

In my own case as a student in an office, and in the single instance of my being a preceptor to a student in my office, the experiences were gratifying and, I believe, thoroughly profitable to all concerned; but in each case it was understood in advance that with all reasonable celerity the advantages of the school were to be embraced and the degree attained. But in multitudes of cases of students in offices the arrangement has not yielded the most profitable results. The preceptor, through incapacity or inattention, has failed to do his duty in wisely and judiciously superintending and pushing the studies of the learner, while on his part the learner, even with the best desires and intentions, has been incapable of rendering the principal such help as he would have been glad to have. In proportion as the student becomes advanced and experienced, wherever that progress has been made, the profitableness of the office relation increases. The advanced student is more apt to learn from what he sees, and he can render in return more efficient service.

This leads me to say that in every practice whose volume justifies considerable assistance, that assistance may be best rendered by one who has had the teaching and the training evidenced by the possession of the degree. For nineteen years I have had in my office graduate assistance. In succession four different men have occupied the position. With myself, there have been five parties in interest. According to the best of my knowledge and belief, there has been no time when any one of the five would not have testified that the arrangement had been a profitable one for him. By such plan each party gets the best end of the bargain, while the patronage of the office is more promptly and better served. The principal is relieved from demands which he could not possibly meet, and this surplus which his attainments and reputation have attracted, instead of being driven away, remains to be acceptably served under the same roof, with his advice whenever needed, and in a way to yield to him some return,—a fair proportion.

The assistant is presumably a recent graduate, one who has his name and fame to make. I believe the young dentist at the time of obtaining his degree is more practically educated—that is, he is better fitted for actually doing his work—than is the young man in most of the other professions and specialties at the time of receiving his degree. Of course, the young dentist has still much to learn from experience,—I suppose most of us would say as much after graduation as before; but the principal thing which the young graduate needs is a chance to go to work, the opportunity to show what he can do, to inspire confidence and to become known. This the assistant's position gives him. Being vouched for by one whose recommendation is reliable and influential, his work begins at once, his income begins at once; and idleness and waiting, the proverbial starvation period, are eliminated. He gets compensation in money, he gets it in constant conference, aid, and counsel from the older and more experienced man, and he gets it in a growing and developing practice for himself which the transfers of his principal and his own faithful competency are all the time building up.

This assistant, of whom I have just made mention, should be, like his principal, an all-round man, capable of doing with his own hands everything, operative and mechanical, within the usual range of a mixed dental practice. Particularly should this be the case if the volume of the business of the office is sufficient for the employment of only one assistant; although there are instances most satisfactory in their general working, of the association together of two men, one of whom, from taste and capacity, gives his attention to the operative department, while the other, for like reasons, attends to the prosthetic branch.

During a large part of the time for the last twelve years I have had regularly a second assistant, whose work has been entirely or almost entirely in the laboratory. The intent is to have the person in the laboratory do the laboratory work, the whole laboratory work and nothing but the laboratory work. During these dozen years the place has been filled by a succession of different individuals with differing qualifications, but usually with a good degree of competence for the work. Several times the place has been occupied by a young man who had had one or two years in a dental college, and who found it convenient to spend a summer vacation or a longer interval in earning some money, while at the same time adding to his store of knowledge and experience. Two young men were of the kind giving their whole attention to laboratory work. One of these was a young Englishman, who had been regularly through a portion of the long apprenticeship prescribed in his native land, and the other was a young colored man. Each one of these was particulary capable in his work. The young colored man of good abilities of the right sort may find in the dental laboratory full, congenial, and remunerative employment in any community where there is the work to be done; and he may thus make a success in locations where he could hope for little as an operator or as a general practitioner.

To fill the laboratory man's place it is far from necessary that one be a dentist. Though it is well for him to know something of anatomy, physiology, and pathology, he is likely to be more helped in his work by a knowledge of physics, chemistry, metallurgy, and the principles of force and mechanics. One who has served an apprenticeship to the jeweller's trade will usually be found to have special aptness in neatly and ingeniously constructing the varied appliances needed to meet in the best way the requirements for regulation and replacement.

For nine months I have had in my laboratory a young woman, and for six months, with such occasional little assistance as the others of the office force could render, she has done alone my laboratory work, and also, as she had opportunity, some work in the same line for several of my dental neighbors. Quite a large part of her teaching during the first three months was by a proficient young man who had had two years in the Harvard school, and who spent the summer vacation in the laboratory with her. Until she came to the laboratory she had not had practice in any similar occupation, and, as would be expected, her present capacity for the despatch of the work is not what it will become with more experience. She is most careful, painstaking, and earnestly scrupulous in seeking to avoid chances for error and failure. She began the work with the avowed purpose to make it a permanent occupation; and I must testify that the experience thus far has served to confirm my long-held conviction that this field, in which women hitherto have done so little, may be and, doubtless, in the future will be one in which they will appropriately and helpfully do much.

While I am not, in a certain sense of the term, a woman's rights advocate, I am an earnest believer in the idea that any woman should have the right, does have the right, to engage in any worthy occupation for the pursuit of which she is capable, and she has the right to proper compensation for work which she does. As a worker she is entitled to no whit less respect, but rather the reverse. In our section of the country there are very many more women than men. Of these women a large proportion are so circumstanced that they must be dependent upon others for support or they must do something to help themselves. How infinitely more honorable is the spirit of self-helping independence.

During a good many years I had an office-boy, a succession of office-boys, whose services, while not always most acceptable in manner, were a real help. The office-boy is still retained, but for several years his duties have been entirely outside of the office.

One of the most conveniently useful members of the office *personnel* is the office young lady. She comes in the morning and sees that the rooms are aired and dusted, and everything neat and in order, and so kept throughout the day. She sees all the patients as they come in, learns the objects of the calls, makes appointments, and transacts with callers all the business which she can. She assists ladies and children in removing their wraps, and seeks to contribute to the comfort of all. She prepares ready for introduction into the cavity filling-materials of various kinds. She assists in the application of the rubber dam or in the use of other agencies for maintaining dryness. She holds the rubber beyond the border of a far-reaching cervical cavity, she aids in the starting of fillings, she anneals gold and passes it to the position where it is to be packed, or with the hand-mallet consolidates it; and she does numberless things to help on the progress of an operation. However much we may pride ourselves on our ability to perform any operation unaided, there is no denying that in many operations our own comfort and that of the patient may be far better served by two pairs of hands than by one. After every patient the young lady sees that all instruments and appliances are cleaned and restored to their places before she summons the next patient. She should do as much as possible about recording the cases, keeping the books, sending and receipting the bills, and looking after a portion of the office correspondence. For more than a year I have been a good deal helped in my correspondence by the use of a phonograph and a type-writer, the letter being spoken by me to the phonograph, and the young lady and the machines doing the rest. The young lady attends to the telephone, and is the general means of communication about the office, and, as far as possible, between the office and the outside world. She is engaged with the understanding that she is to make herself useful in every possible way about the office.

A practitioner of dentistry who has always worked by himself can have little idea how convenient and how comfortable a thing it is to have such assistance as I have just named. The presence of a young lady about the office, instead of being looked on by patients as an intrusion, is regarded by them as a pleasant thing, and they are quick to appreciate the shortening of operations and the other contributions to their comfort which she is able to make.

I am not proficient at all in the science of economics, but I be lieve there are sound principles underlying such combinations of workers as I have described. Not all portions of the work in a dental office require the skill of the specially trained and experienced practitioner. When the demands upon the office become more than the dentist alone can meet, it is wise in him to delegate to other hands that which other hands may do. He may thus retain and conduct a practice of a volume otherwise impossible, receiving therefor a compensation which is shared with those who are given employment, and who share in the service. Rightly managed, the plan is a consolidation of interests to the advantage of all,—those who serve and those who are served.

